# Molybdenum Disulfide-Based Nanoprobes: Preparation and Sensing Application

**DOI:** 10.3390/bios12020087

**Published:** 2022-01-31

**Authors:** Lingbo Gong, Lin Feng, Youwei Zheng, Yi Luo, Dan Zhu, Jie Chao, Shao Su, Lianhui Wang

**Affiliations:** State Key Laboratory of Organic Electronics and Information Displays & Jiangsu Key Laboratory for Biosensors, Institute of Advanced Materials (IAM), Nanjing University of Posts and Telecommunications, 9 Wenyuan Road, Nanjing 210023, China; gonglingboo@163.com (L.G.); kitten_fl@icloud.com (L.F.); m15884818698@163.com (Y.Z.); iamyluo@njupt.edu.cn (Y.L.); iamdzhu@njupt.edu.cn (D.Z.); iamjchao@njupt.edu.cn (J.C.)

**Keywords:** molybdenum disulfide, nanoprobe, signal amplification, sensor, detection

## Abstract

The use of nanoprobes in sensors is a popular way to amplify their analytical performance. Coupled with two-dimensional nanomaterials, nanoprobes have been widely used to construct fluorescence, electrochemical, electrochemiluminescence (ECL), colorimetric, surface enhanced Raman scattering (SERS) and surface plasmon resonance (SPR) sensors for target molecules’ detection due to their extraordinary signal amplification effect. The MoS_2_ nanosheet is an emerging layered nanomaterial with excellent chemical and physical properties, which has been considered as an ideal supporting substrate to design nanoprobes for the construction of sensors. Herein, the development and application of molybdenum disulfide (MoS_2_)-based nanoprobes is reviewed. First, the preparation principle of MoS_2_-based nanoprobes was introduced. Second, the sensing application of MoS_2_-based nanoprobes was summarized. Finally, the prospect and challenge of MoS_2_-based nanoprobes in future were discussed.

## 1. Introduction

As a powerful tool, a sensor has been employed to analyze chemical/biological molecules coupled with different detection methods, such as fluorescence, electrochemistry, electrochemiluminescence (ECL), colorimetry, surface enhanced Raman scattering (SERS) and surface plasmon resonance (SPR). To improve the analytical performance, many signal amplification strategies have been introduced into the construction of sensors, including DNA amplification technology, DNA walker, enzyme-assisted signal amplification and nanoprobes [1,2,3,4,5]. With the rapid development of nanomaterials, the nanoprobe has been considered as a promising signal amplification strategy to improve the performance of sensors.

Since gold nanoparticles (AuNPs) were introduced into the construction of nanoprobes [6,7], different kinds of nanomaterials have been extensively employed to construct nanoprobes due to their high surface area, excellent electrical and optical properties, high catalytic ability, excellent chemical stability and easy functionalization [8,9,10,11,12], such as noble metal nanoparticles [13,14], metal oxides [15], graphene and its derivative [16,17], transition metal dichalcogenides [18,19,20], and so on. The outstanding properties of nanomaterials allowed nanoprobes to easily load a large number of recognition and signal units, which can efficiently amplify the detection signal. Furthermore, the high biocompatibility of nanoprobes paves a way to analyze target molecules in vivo.

**Figure 1:**
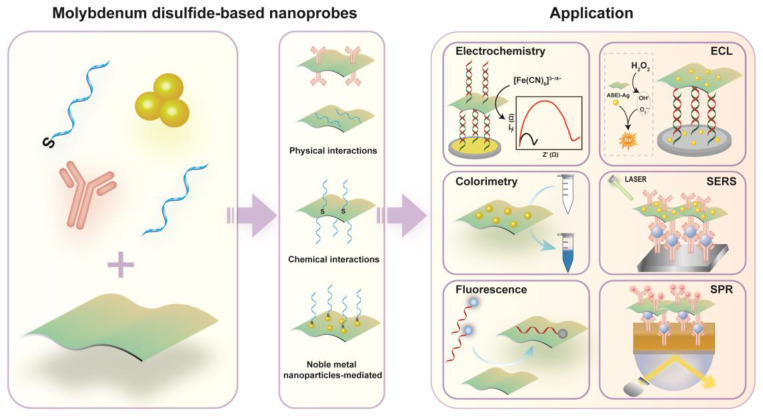
Schematic diagram of preparation and sensing application of molybdenum disulfide-based nanoprobe.

MoS_2_ is an emerging material star, which is a member of transition metal dichalcogenides. Due to its typical graphene-like layered nanostructure, MoS_2_ is also a potential candidate to construct the ideal nanoprobe due to its unique physical, chemical, and electronic properties, such as a large surface area, high conductivity, excellent quenching activity, accepted Raman enhancement effect and easy functionalization [21]. The recognition units or signal units assembled onto the MoS_2_ nanosheet to form MoS_2_-based nanoprobes, which exhibited a high molecular recognition ability, excellent chemical stability, accepted biocompatibility and a strong signal amplification effect. Moreover, MoS_2_-based nanoprobes easily coupled with other signal amplification strategies to further amplify detection performances, including sensitivity, selectivity, reproducibility, stability, etc. Inspired by the rapid development of MoS_2_-based nanoprobes in sensing application, it is necessary to summarize its exciting advances (Figure 1). From this review, we hope to offer some useful suggestions to new researchers in the sensing field.

## 2. Preparation of MoS_2_-Based Nanoprobes

Generally, a MoS_2_ nanosheet can load chemical/biological recognition units and signal molecules to form a nanoprobe via physical adsorption, chemical bond and noble metal-mediated methods, respectively [22]. It should be noted that MoS_2_-based nanoprobes prepared by different methods exhibited different advantages and disadvantages, which is listed in Table 1. According to the sensing application, the suitable nanoprobe coupled with analytical techniques often brings a better analytical performance, such as higher sensitivity, better selectivity and longer storage stability.

### 2.1. Physical Interaction

A MoS_2_ nanosheet possesses a graphene-like layered nanostructure with a large surface area. As a result, it is easy to nonspecifically adsorb chemical or biological molecules via van der Waals force and electrostatic interactions. Notably, a MoS_2_ nanosheet also exhibits different affinity towards single-strand (ss) and double-strand (ds) DNA. Based on these properties, MoS_2_-based nanoprobes including DNA-MoS_2_, aptamer-MoS_2_ and peptide-MoS_2_ probes, have been designed. For example, Zhu et al. firstly developed a fluorescence sensing platform by adsorbing DNA on the surface of a MoS_2_ nanosheet as a nanoprobe [23]. A general platform for the construction of sensors was developed by combining the different affinity of the MoS_2_ nanosheet towards ssDNA and dsDNA with its high fluorescence quenching efficiency. Five years later, Zhu and co-workers explored the possibility to construct MoS_2_-based fluorescence nanoprobes by adsorbing hairpin DNA [24]. Besides DNA, rhodamine B isothiocyanate (RhoBS) and antibodies also can be loaded on the surface of the MoS_2_ nanosheet to form nanoprobes via physical adsorption and hydrophobic interactions, which can be used to determine silver ions and Escherichia coli by fluorescence and the SPR method, respectively [25,26].

### 2.2. Chemical Interaction

Recognition and signal units assembled on the MoS_2_ surface via chemical interaction is another efficient way to form MoS_2_-based nanoprobes. A popular method is to bind recognition and signal units with MoS_2_ via classical thiol-metal coordination (typical Mo-S coordination). A typical example was given by Li et al., who designed a MoS_2_-based fluorescence nanoprobe for caspase-3 activity detection and images of cell apoptosis by efficiently conjugating two peptides with polydopamine-decorated MoS_2_ nanosheets [28]. Since poly-cytosine (poly-C) DNA was proved as a high-affinity ligand for 2D nanomaterials [37], Xiao et al. [29] constructed a MoS_2_-based nanoprobe by assembling poly-C-modulated diblock molecular beacons on the MoS_2_ surface. Experimental results suggested the length of poly-C could efficiently affect the analytical performance of the nanoprobe due to the regulation of the surface density [29].

### 2.3. Noble Metal Nanoparticles-Mediated 

As we know, noble metal nanoparticles have excellent advantages, including high catalytic activity, high electrical conductivity, large surface area and excellent biocompatibility, which have been widely used in sensing fields [38,39]. MoS_2_ nanosheets have been proved as an ideal substrate to hybridize with noble metal nanoparticles [40,41]. As a result, the synergistic effect of noble metal nanoparticle-decorated MoS_2_ nanocomposites brings faster electron transfer, higher catalytic activity, higher quenching efficiency and larger loading capacity, which have been considered as promising candidates to construct a nanoprobe. As a result, the designed nanoprobe not only retains the inherent characteristics of the hybrid element, but also brings better performance and enlarges its application fields. For instance, Su and co-worker prepared AuNP-decorated MoS_2_ nanocomposites (MoS_2_-AuNPs) to construct electrochemical nanoprobes for biological molecules’ detection with accepted results due to the signal amplification [30,32]. The recognition and signal units can efficiently co-immobilize on the MoS_2_ surface via noble metal-mediated nanoparticles, such as an Au-S bond. Inspired by these exciting results, other noble metal nanoparticles were also successfully supported on the surface of molybdenum disulfide to construct a high-performance nanoprobe for sensing application [42,43,44].

## 3. MoS_2_-Based Nanoprobes for Sensing Applications

MoS_2_-based nanoprobes can efficiently amplify the analytical performance due to their large loading amount, excellent electron transfer ability, high fluorescence quenching ability, and high Raman enhancement effect. As we know, different detection methods possess their inherent advantages and disadvantages (Table 2). Therefore, MoS_2_-based nanoprobes coupled with suitable analytical methods is a best way to construct sensors for obtaining high-performance target molecules’ detection. Herein, the recent progresses of MoS_2_-based nanoprobes coupled with electrochemical, ECL, colorimetric, SERS, fluorescence, and SPR methods is summarized (Table 3).

### 3.1. Electrochemical Sensors

**Figure 2:**
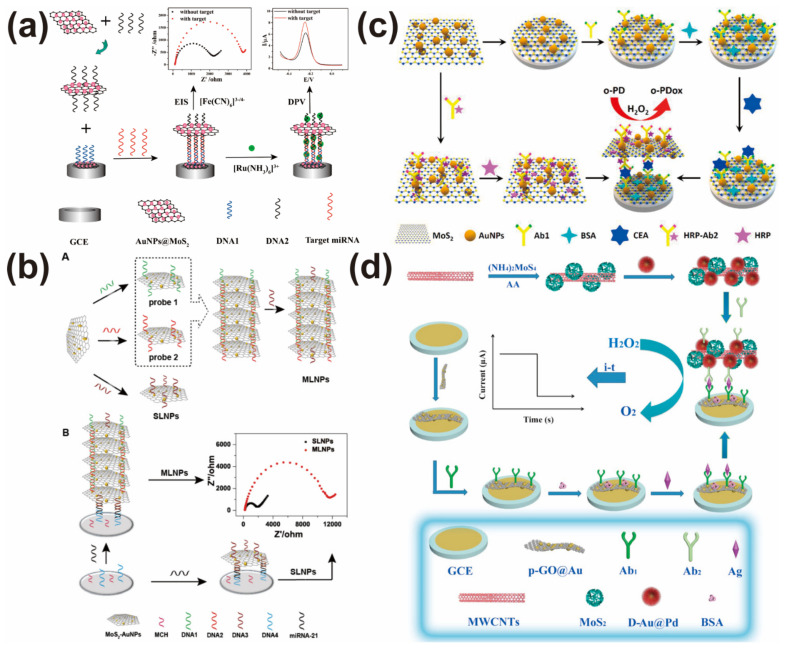
(**a**) Construction of dual-mode electrochemical sensor for miRNA-21 detection based on MoS_2_-based nanoprobes. Reprinted with permission from [32]. Copyright 2017, Elsevier. (**b**) Construction of multilayer MoS_2_-based nanoprobes for miRNA-21 analysis. Reprinted with permission from [46]. Copyright 2020, Royal Society of Chemistry. (**c**) Illustration of electrochemical immunosensor by coupling MoS_2_-based nanoprobe with triple signal amplification. Reprinted with permission from [31]. Copyright 2019, Elsevier. (**d**) Construction and application of MoS_2_-based nanoprobes for electrochemical analysis of HBeAg. Reprinted with permission from [48]. Copyright 2017, Elsevier.

MoS_2_-based nanoprobe is a promising candidate to construct electrochemical sensors due to its high conductivity and high loaded capacity. To further improve the electronic properties of MoS_2_-based nanoprobes, the introduction of noble metal nanoparticles into nanoprobes has become a popular method. Therefore, gold nanoparticles (AuNPs), platinum nanoparticles (PtNPs), silver nanoparticles (AgNPs), and Au@AgPt nanocubes have been selected to form MoS_2_-based nanocomposites, which were further used to construct high-performance nanoprobes. For example, Su et al. used AuNPs-decorated MoS_2_ nanocomposites to construct nanoprobes [32]. They utilized [Fe(CN)_6_]^3−/4−^ and [Ru(NH_3_)_6_]^3+^ as signal molecules to design a dual-mode electrochemical sensor for microRNA-21 (miRNA-21) detection. As shown in Figure 2a, the MoS_2_-based nanoprobes can efficiently amplify electrochemical responses by differential pulse voltammetry (DPV) and electrochemical impedance spectroscopy (EIS). Notably, the detection limit of this sensor obtained from EIS (0.45 fM) is lower than that obtained from DPV (0.78 fM), which is ascribed to the unique properties of 2D nanoprobes. This exciting finding opened a new way to construct electrochemical sensors. After three years, the same group developed a MoS_2_-based multilayer nanoprobe by using a DNA hybridization reaction (Figure 2b). Compared with a classical MoS_2_-based single-layer nanoprobe, the designed electrochemical sensor showed an ultrawide dynamic range (10 aM-1 μM) and ultralow detection limit (38 aM) for miRNA-21 detection. The big structure of a MoS_2_-based multilayer nanoprobe and a large amount of negative DNA loaded on a multilayer nanoprobe both greatly hindered the electron transfer between [Fe(CN)_6_]^3−/4−^ and the electrode surface, leading to the impedance value of this sensor obviously increasing with the addition of trace miRNA-21 [46]. To further amplify the detection performance, Bai’s group coupled a MoS_2_-based nanoprobe with enzyme-assisted target recycling amplification to sensitively analyze the Sul1 gene. Due to the synergistic effect of two amplification strategies, the developed electrochemical sensor can determine 29.57 fM Sul1 gene with high selectivity [47]. Similarly, Ji et al. designed an electrochemical sensor for Pb^2+^ analysis based on a MoS_2_-based nanoprobe and hemin/G-quadruplex DNAzyme [33]. The specificity of a DNAzyme combined with the high conductivity of MoS_2_-AuPt nanocomposites means this sensor has a lower detection limit for Pb^2+^ analysis (38 fg mL^−1^).

A MoS_2_-based nanoprobe has been also employed to construct electrochemical immunosensors. For example, Li et al. constructed an immunosensor by using CeO_2_-MoS_2_-Pb^2+^-Ab_2_ as a signal probe [36]. Ingeniously, Pb^2+^ can adsorbs and aggregates on the surface of a CeO_2_-MoS_2_ nanocomposite, which can not only anchor antibodies, but also generate and enhance electrical signals. This novel design of a MoS_2_-based nanoprobe achieved the purpose of the sensitive detection of CEA. To further improve the analytical performance, Su et al. [31] constructed an enzyme-assisted signal amplification strategy for carcinoembryonic antigen (CEA) analysis by taking the advantages of MoS_2_-AuNPs nanocomposites and the catalytic activity of enzymes (Figure 2c). In this work, MoS_2_-AuNPs can not only accelerate electron transfer due to its high conductivity, but also can load a large number of enzymes and antibodies to achieve multiple signal amplification. Therefore, the proposed immunosensor detected down to 1.2 fg mL^−1^ CEA with high selectivity and good stability. Similarly, Gao et al. developed a signal probe by combining gold@palladium nanoparticle-loaded molybdenum disulfide with multi-walled carbon nanotubes (Au@Pd/MoS_2_@MWCNTs) to efficiently analyze the hepatitis B e antigen (HBeAg) [48]. With the addition of HBeAg, a classical sandwich immunosensor was formed (Figure 2d). The introduced signal probe contained Au@Pd nanoparticles, which can efficiently catalyze hydrogen peroxide (H_2_O_2_) to generate high electrochemical signal. Therefore, the sensor got a low detection limit of 26 fg mL^−1^ with the help of signal probe amplification. Other MoS_2_-based electrochemical nanoprobes were also used to detect cardiac troponin I, HBsAg, and CEA due to their outstanding signal amplification effect, respectively [49,50,74].

### 3.2. ECL Sensors

**Figure 3:**
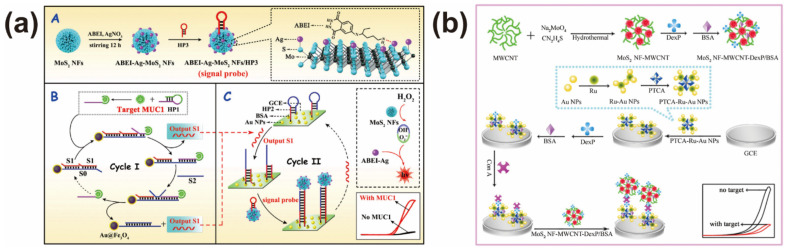
(**a**) Development of ECL biosensor for mucin 1 analysis based on MoS_2_-based nanoprobe. Reprinted with permission from [52]. Copyright 2018, American Chemical Society. (**b**) Illustration of ECL biosensor for concanavalin detection A by using the high-efficient quenching ability of MoS_2_ nanoflower. Reprinted with permission from [54]. Copyright 2017, Elsevier.

A few layers of MoS_2_ the nanosheet possess a direct bandgap and a large surface. These properties made the MoS_2_-based nanoprobe a potential candidate to construct electrochemiluminescence (ECL) sensors. Usually, a MoS_2_-based nanoprobe is used as a co-reaction promoter to efficiently amplify the detection signal, called a “signal-on” detection mechanism. An example was offered by Li et al., who constructed a ECL sensor for mucin 1 (MUC1) analysis by coupling a target recycling signal amplification strategy and a MoS_2_-based nanoprobe [52]. The prepared MoS_2_ nanoflowers can heavily load N-(aminobutyl)-N-(ethylisoluminol) (ABEI)-decorated AgNPs as signal amplifiers, which can catalyze ABEI-H_2_O_2_ to improve the detection intensity. As shown in Figure 3a, the added MUC1 triggered the signal amplification process, leading to the designed ECL aptasensor having a wide linear range (1 fg mL^−1^ to 10 ng mL^−1^) and low detection limit (0.58 fg mL^−1^) for MUC1 determination. Another ECL MoS_2_-based nanoprobe was constructed by MoS_2_@Au nanocomposites [53]. With the assistance of exonuclease III-driven DNA walker, a sensitive ECL sensor was developed for 8.9 pM sialic acid-binding immunoglobulin (Ig)-like lectin 5 analysis.

A MoS_2_-based nanoprobe was also used to construct “signal off” ECL sensors by utilizing the high quenching ability of MoS_2_ nanostructures. For example, Yuan and co-worker reported a ECL sensor for concanavalin A (Con A) determination according to the signal-off sensing mechanism [54]. The as-prepared MoS_2_ nanoflowers highly quenched the ECL signal of the Ru complex, making the ECL response decrease with the increasing ConA concentration, ranging from 1.0 pg mL^−1^–100 ng mL^−1^ (Figure 3b). According to the quenching properties of MoS_2_-based nanoprobes in ECL sensing application, several ECL sensors were constructed for beta-amyloid (Aβ), CA19-9 antigen and human epididymal specific protein 4 detection, respectively [55,56,57]. All experimental data suggested the introduction of MoS_2_-based nanoprobes can efficiently improve the analytical performances, such as linear range, detection limit, analytical time, etc.

### 3.3. Colorimetric Sensors

**Figure 4:**
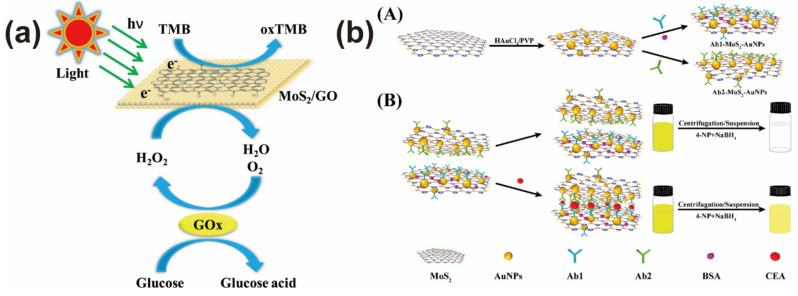
(**a**) Colorimetric analysis of glucose by coupling peroxidase-like MoS_2_-based nanoprobe and glucose oxidase. Reprinted with permission from [59]. Copyright 2016, Elsevier. (**b**) Construction of a MoS_2_-based colorimetric biosensor for carcinoembryonic antigen analysis. Reprinted with permission from [30]. Copyright 2018, American Chemical Society.

Previous works proved that MoS_2_ nanostructures have peroxidase mimicking activity with high chemical and thermal stability [74]. For example, Zhao et al. found that sodium dodecyl sulfate-conjugated MoS_2_ nanoparticles (SDS-MoS_2_ NPs) can efficiently catalyze a 3,3,5,5-tetramethylbenzidine (TMB) and hydrogen peroxide (H_2_O_2_) reaction strategy, exhibiting peroxidase-like activity for the detection of glucose [75]. To improve the peroxidase-like activity of MoS_2_ nanostructures, the formation of MoS_2_-based nanocomposites is a universal method. These nanocomposites offer the opportunity to develop high-performance colorimetric nanoprobes due to their better catalytic activity, such as MoS_2_-carbon nanotubes [76], MoS_2_-g-C_3_N_4_ [58], MoS_2_-graphene oxide [59], MoS_2_-Au@Pt [77], etc. According to this concept, Peng et al. used a MoS_2_-graphene oxide (MoS_2_-GO) nanocomposite instead of a biological enzyme to colorimetricly detect H_2_O_2_ and glucose [59]. The synergistic effect of MoS_2_ and graphene oxide made this designed colorimetric sensor analyze H_2_O_2_ and glucose in serum samples by the naked-eye (Figure 4a). Compared with graphene, noble metal nanostructures hybridized with a MoS_2_ nanosheet can bring outstanding peroxidase-like activity. A typical example was offered by Su and co-workers, who designed a colorimetric sensor for cysteine analysis based on a MoS_2_-Au@Pt nanoprobe [77]. The enzyme-mimicking activity made this sensor show a wide linear range and low detection limit for cysteine detection. Moreover, this colorimetric sensor can determine cysteine in medical tables. Similarly, Singh et al. utilized the highly-efficient peroxidase-like activity of Fe-doped MoS_2_ nanomaterials to colorimetricly detect glutathione in buffer and human serum [34]. The satisfactory results further proved the excellent application of MoS_2_-based nanoprobes in the colorimetric sensing field.

Besides peroxidase-like activity, another reason for MoS_2_-based nanocomposites in the colorimetric sensing application is the high catalytic activity. By utilizing this property, Su et al. constructed a colorimetric nanoprobe by assembling an anti-CEA on the surface of a MoS_2_-AuNPs nanocomposite [30]. The assembled amount of anti-CEA greatly influenced the catalytic activity of the MoS_2_-AuNPs nanocomposite, which can be used to recognize and detect CEA by catalyzing the reaction of 4-nitrophenol (4-NP) and sodium borohydride (NaBH_4_). Corresponding with the solution color and adsorption intensity, the developed can analyze 5 pg mL^−1^–10 ng mL^−1^ of CEA with high selectivity (Figure 4b). This potential colorimetric sensing application inspired more researchers to synthesize different kinds of MoS_2_-based nanocomposites with high catalytic activity, such as AuNP or PtNP decorated Ni promoted MoS_2_ nanocomposites [78], multi-element nanocomposites composed by noble metal nanoparticles, polyaniline microtubes, and Fe_3_O_4_ and MoS_2_ nanosheets [79].

### 3.4. SERS Sensors

**Figure 5:**
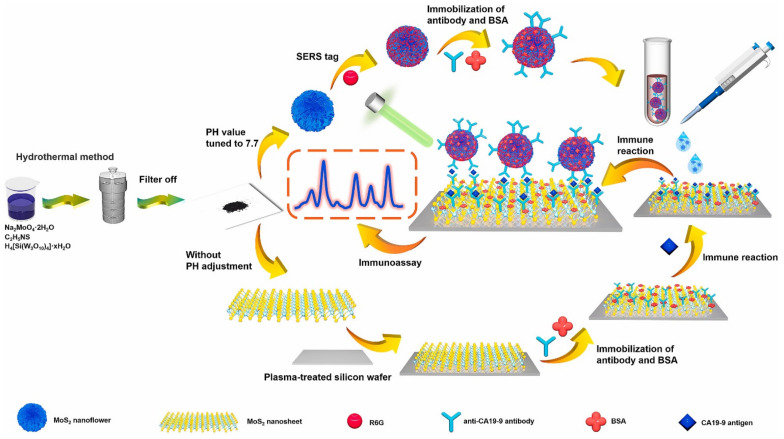
Illustration of MoS_2_-based immunosensor for CA19-9 detection. Reprinted with permission from [64]. Copyright 2021, Elsevier.

As a graphene-like 2D layered nanomaterial, a MoS_2_ nanosheet also exhibits an excellent Raman enhancement effect due to the chemical enhancement mechanism [80]. Decoration with noble metal nanoparticles, the synergistic effect of chemical enhancement and electromagnetic enhancement makes the MoS_2_-noble metal nanoparticles’ nanohybrids exhibit a better Raman enhancement effect. Therefore, MoS_2_ and its nanocomposites are often employed as SERS-active substrates to construct sensors for target molecules’ detection [81,82]. Besides SERS-active substrates, MoS_2_-based nanohybrids have also been used to construct nanoprobes for sensing application. For example, Jiang et al. [64] developed a MoS_2_-based immunosensor for the carbohydrate antigen 19-9′s (CA19-9) detection by using a MoS_2_ nanosheet as a SERS-active substrate and a MoS_2_ nanoflower as a SERS tag (Figure 5). Expectedly, this sandwich design exhibited a desirable enhancement effect on CA19-9 analysis, resulting in a wide linear range (5 × 10^−4^–1 × 10^2^ IU·mL^−1^) and low detection limit (3.43 × 10^−4^ IU·mL^−1^). More meaningfully, this designed immunosensor showed accepted results for CA19-9 detection in clinical patient serum samples, which was in agreement with the conventional chemiluminescent immunoassay. Similarly, Medetalibeyoglu et al. also reported a sandwich-type immunosensor for CEA detection by using 4-mercaptobenzoic acid assembled AuNPs-decorated MoS_2_ nanoflowers (MoS_2_ NFs@Au NPs/MBA) as SERS tag [65]. Coupled with Ti_3_C_2_T_x_ MXene-based SERS-active substrate, this immunosensor detected as low as 0.033 pg mL^−1^ of CEA, with high selectivity, stability and repeatability. More interestingly, a MoS_2_-based SERS nanoprobe is also a powerful tool for label-free SERS imaging. For example, Fei et al. offered an example of a MoS_2_-based nanoprobe for SERS imaging in living 4T1 cells [66]. Experimental results suggested that a MoS_2_-based nanoprobe may be the promising alternative because of its intrinsic vibrational bands in the Raman-silence region of cells.

### 3.5. Fluorescence Sensors

**Figure 6:**
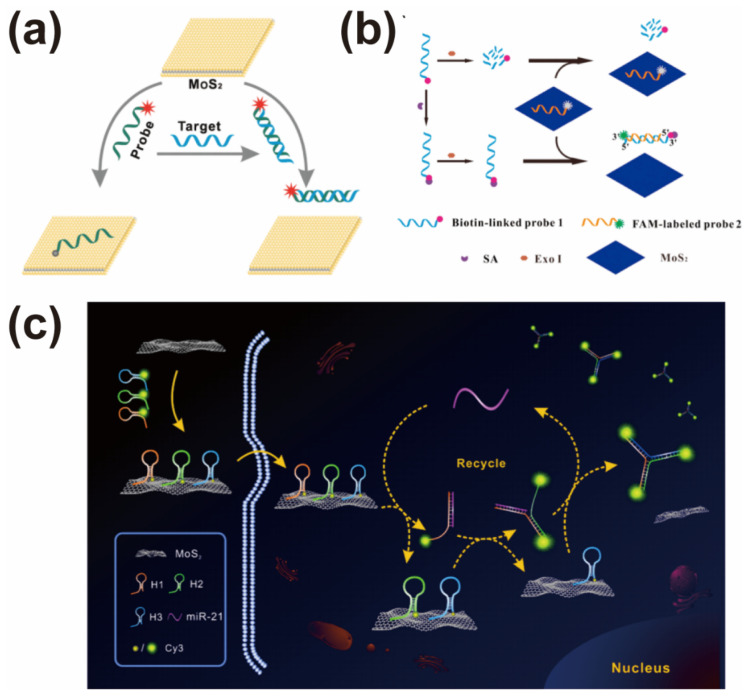
(**a**) Cartoon of MoS_2_-based fluorescence sensor for DNA detection. Reprinted with permission from [23]. Copyright 2013, American Chemical Society. (**b**) Illustration of Exo III-assisted fluorescence biosensor for streptavidin detection based on MoS_2_-based nanoprobe. Reprinted with permission from [69]. Copyright 2015, Elsevier. (**c**) MoS_2_-based nanoprobe coupled with signal amplification strategy for ultrasensitive detection and imaging of miRNA-21 expression in living cells. Reprinted with permission from [95]. Copyright 2019, American Chemical Society.

The tunable layer thickness of the MoS_2_ nanosheet leads to its indirect to direct band-gap transition, which generates excellent optical properties. Especially, the outstanding quenching ability towards organic dyes suggests that a MoS_2_ nanosheet can be employed as a nanoquencher to construct fluorescence sensors. Zhu et al. had given a first example of a fluorescence sensor for targetting DNA and other small molecules by using a MoS_2_ nanosheet as a sensing probe [23]. The different affinity of the MoS_2_ nanosheet towards ssDNA and dsDNA makes the labeled 5-carboxyfluorescein (FAM) close to or far from the surface of the MoS_2_ nanosheet, resulting in the fluorescence signal recovering with the formation of dsDNA (Figure 6a). This exciting finding inspired more and more researchers to develop fluorescence sensors for target molecules’ detection by using MoS_2_-based sensing nanoprobes. A typical design is coupling an aptamer with a MoS_2_-based nanoprobe to analyze nucleic acids, proteins, thrombin, metal ions, kanamycin, ochratoxin A, and so on [67,83,84,85]. For example, Kong et al. utilized the high-efficient quenching ability of a MoS_2_ nanosheet to develop a fluorescence sensor for prostate specific antigen (PSA) analysis [68]. The structure of the aptamer was changed with the recognition of the PSA, leading to the aptamer-PSA product releasing from the MoS_2_ nanosheet and the fluorescence recovering. Under optimal conditions, this designed sensor can detect as low as 0.2 ng mL^−1^ of PSA with high selectivity.

To further improve the analytical performance, several signal amplification strategies coupled with MoS_2_-based nanoprobes were introduced into the construction of fluorescent sensors. For example, Xiang et al. reported a fluorescence sensor for streptavidin (SA) detection by coupling exonuclease III (Exo III)-assisted DNA recycling amplification with MoS_2_-based nanoprobes [69]. As shown in Figure 6b, probe 1 was not degraded by Exo III because of the binding of SA and biotin. Subsequently, the protected probe 1 hybridized with probe 2, which can be digested by Exo III. The continually released FAM led to a strong fluorescence signal due to the signal amplification, producing a low detection limit of 0.67 ng mL^−1^ for SA detection. Similarly, Xiao et al. combined duplex-specific nuclease (DSN)-mediated signal amplification with MoS_2_-based nanoprobes to develop a fluorescence for microRNA (miRNA) detection [24]. In the presence of miRNA, molecular beacons adsorbed onto the MoS_2_ nanosheet changed to DNA–RNA heteroduplexes and were released from the MoS_2_ nanosheet due to the hybridization reaction. The formed DNA–RNA heteroduplexes were digested by the DSN and the target miRNA was released to trigger the next hybridization reaction. Under optimal conditions, this sensor showed a wide dynamic range (10 fM–10 nM), low detection limit (10 fM) and high selectivity for let-7a analysis. In the same year, Xiao et al. also constructed a poly-cytosine (poly-C)-mediated MoS_2_-based nanoprobe coupled with a DSN signal amplification strategy for miRNA detection [29]. The introduction of a unique poly-C tails design led to a lower detection limit (3.4 fM) than classical molecular beacon-loaded MoS_2_-based nanoprobes. Other signal amplification strategies have also been introduced into the construction of fluorescence sensors based on MoS_2_-based nanoprobes, such as catalytic hairpin assembly (CHA), a hybrid chain reaction (HCR), rolling circle amplification (RCA), etc., [86,87,88,89,90].

A MoS_2_-based fluorescence nanoprobe is also a potential tool for the detection of intracellular biomolecules due to its excellent biocompatibility, such as ATP, microRNA, etc., [91,92,93]. For example, Ju and co-worker assembled a chlorine e6 (Ce6) labelled ATP aptamer onto a MoS_2_ nanoplate to develop an intracellular nanoprobe for ATP detection and imaging based on the favorable biocompatibility [94]. It was noted that this designed MoS_2_-based nanoprobe not only sensitively and selectively analyzed ATP in living cells, but also could achieve controllable photodynamic therapy. Inspired by this exciting work, Li et al. immobilized two peptides onto a polydopamine (PDA)-functionalized MoS_2_ nanointerface to construct a fluorescence nanoprobe for caspase-3 activity detection [28]. Caspase-3 was activated with the cell apoptosis, leading to the cleavage of a peptide labeled with fluorescence dye and the trigger of “turn on” fluorescence imaging. According to this design, the developed fluorescence biosensor showed a lower detection limit of 0.33 ng mL^−1^ compared with some previous reports. For the purpose of trace biomolecules analysis, Zhu et al. developed an ultrasensitive fluorescence sensor for intracellular miRNA-21 detection and imaging based on MoS_2_ nanoprobes by assembling three Cy3-labelled molecular beacons onto MoS_2_ nanosheets [95]. As shown in Figure 6c, the added miRNA-21 triggered a CHA reaction to form “Y”-shaped DNA structures with multiple Cy3 molecules. This interesting design obtained an ultralow detection limit (75.6 aM) for miRNA-21 detection compared to a general strand displacement-based strategy (8.5 pM). The excellent analytical performance was also proved by the intracellular imaging of miRNA-21 in human breast cancer cells.

### 3.6. SPR Sensors

**Figure 7:**
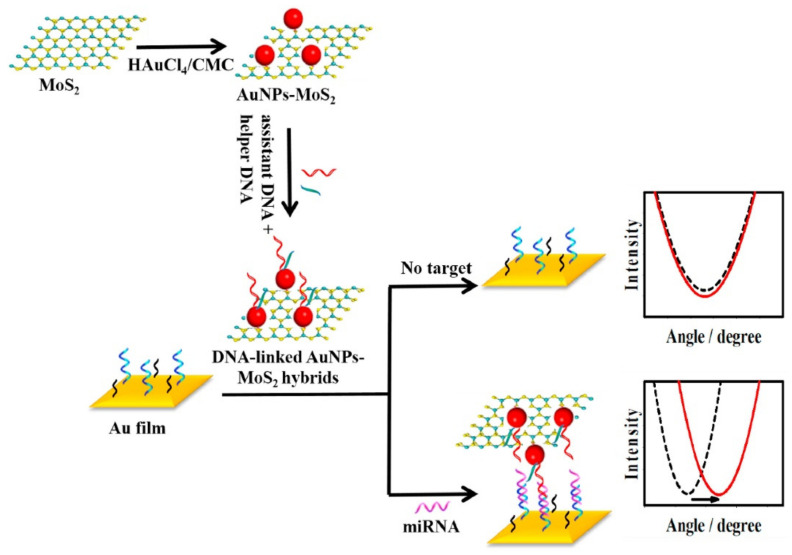
Schematic diagram of SPR biosensor for miRNA-141 detection based on MoS_2_-based nanoprobe. Reprinted with permission from [73]. Copyright 2017, Elsevier.

MoS_2_ and its nanocomposites have been considered as ideal substrates for the construction of SPR sensors due to the unique properties of a MoS_2_ nanosheet, such as high charge carrier mobility and easily functionalization of noble metal nanoparticles [25,96]. As expected, MoS_2_-based SPR sensors are widely used to rapidly, label-free detect biomolecules or real-time and in-situ monitor the biological reaction. For example, Chiu et al. assembled carboxyl-functionalized MoS_2_ sheets (MoS_2_-COOH) onto a gold surface to construct a SPR immunosensor for monitoring a bioaffinity interaction [96]. Experimental data showed that the SPR angles can be amplified by the MoS_2_-COOH chip, which was almost 1.9 folds and 3.1 folds than MoS_2_ and traditional SPR chips when the bovine serum albumin (BSA) concentration was 14.5 nM. Unfortunately, most of the works focused on the development of MoS_2_-based SPR substrates. To explore the potential application of a MoS_2_-based nanoprobe in SPR sensing field, Wang and co-workers developed a SPR biosensor for microRNA-141 (miRNA-141) analysis based on MoS_2_-AuNPs nanocomposites [73]. As shown in Figure 7, a classical sandwich structure was formed in the presence of miRNA-141. The localized plasmon of AuNPs supported onto MoS_2_ nanosheets easily generated the electronic coupling by associating with Au film. As a result, an ultralow detection limit of 0.5 fM for miRNA-141 detection was obtained due to this signal amplification effect. Moreover, this designed SRP biosensor exhibited high selectivity for miRNA-200 family members’ determination.

## 4. Conclusions and Perspective

During the past decade, MoS_2_ as an emerging material has aroused more and more scientists’ interests to construct MoS_2_-based nanoprobes due to its inherent advantages, including the large-scale preparation, tunable bandgap, excellent biocompatibility, easy functionalization with inorganic/organic groups, and outstanding optoelectronic properties. The introduction of MoS_2_-based nanoprobes means sensors coupled with different analytical methods have been successfully employed in environmental monitoring, food safety, biochemical analysis, disease diagnosis, and even homeland safety. With the assistance of MoS_2_-based nanoprobes, the developed sensors exhibited high sensitivity, selectivity, and stability for the detection of chemical and biological molecules. Though great advances in sensing application were obtained, MoS_2_-based nanoprobes still face some challenges in practical application. First, high-quality and large-scale preparation of MoS_2_ nanosheets and their nancomposites should be solved. It is the basic to construct a high-performance MoS_2_-based nanoprobe. The high-quality of the MoS_2_ nanosheet often brings a high-performance MoS_2_-based nanoprobe. Controllable and large-scale preparation of MoS_2_ nanosheets can ensure the repeatability of MoS_2_-based nanoprobes. Second, the recognition unit or signal amplification unit should be efficiently assembled onto the MoS_2_ nanosheet and its nanocomposites. The assembled amount and spatial configuration of the recognition unit or signal amplification unit greatly affects the analytical performance. Third, the preparation mechanism of MoS_2_-based nanoprobes should be further studied. It is important to design a high-efficient nanoprobe for the construction of sensors. Finally, the best combination of the MoS_2_-based nanoprobe and detection method is another important influence parameter for obtaining better analytical performance. We believed that a MoS_2_-based nanoprobe will eventually be used in practical applications in the future with our joint efforts.

## Figures and Tables

**Table 1:** Preparation of MoS_2_-based nanoprobes.

Preparation Mechanism	Advantages	Disadvantages	References
physical interaction	simple, fast, facile, wide variety of binding molecules	unstable	[23,24,25,26,27]
chemical interaction	stable	The binding molecule needs to be modified, few choices of binding molecules	[28,29]
noble metal nanoparticles -mediated	simple, facile, stable, wide variety of binding molecules, properties enhanced	complicated preparation process	[30,31,32,33,34,35,36]

**Table 2:** Comparison of different detection methods.

Detection Method	Advantages	Disadvantages
fluorescence	easy design, simple, versatile, possible quantification	the need of large equipment, poor stability
electrochemical	easy design, simple, fast, facile, quantification, miniaturization	complicated interface design, poor repeatability
electrochemiluminescence	easy design, simple, fast, facile, quantification	complicated interface design, poor reproducibility
colorimetric	simple, facile, no need of equipment	poor sensitivity, poor stability
surface enhanced Raman scattering	fast, high sensitivity, high selectivity, quantification	poor reproducibility, the need of large equipment
surface plasmon resonance	simple, high sensitivity	few application scenarios, the need of large equipment

**Table 3:** MoS_2_-based nanoprobes for sensing applications.

Method	Nanoprobe	Target	Linear Range	LOD	References
Electrochemistry	MoS_2_-AuPt	Pb^2+^	0.1 pg mL^−1^ −1000 ng mL^−1^	38 fg mL^−1^	[33]
	hemin/G-quadruplex-Tb-PdNPs/PDDA-G-MoS_2_	thrombin	0.0001−40 nM	0.062 pM	[45]
	MoS_2_-AuNP	microRNA-21	10 aM–1 μM	38 aM	[46]
	MoS_2_-PANI-Au	Sul1	40 fM–40 nM	29.57 fM	[47]
	Au@Pd/MoS_2_ @MWCNTs	HBeAg	0.1−500 pg mL^−1^	26 fg mL^−1^	[48]
	MoS_2_ NFs/Au@AgPt YNCs	CEA	10 fg mL^−1^ −100 ng mL^−1^	3.09 fg mL^−1^	[49]
	DPCN/MoS_2_	CTnI	10 fg mL^−1^ −100 ng mL^−1^	3.02 fg mL^−1^	[50]
	MoS_2_@Cu_2_O-Au	AFP	0.1 pg mL^−1^ −50 ng mL^−1^	0.037 pg mL^−1^	[51]
ECL	ABEI-Ag-MoS_2_ NFs/HP3	MUC1	1 fg mL^−1^ −10 ng mL^−1^	0.58 fg mL^−1^	[52]
	MoS_2_@Au	Siglec-5	10–500 pM	8.9 pM	[53]
	MoS_2_ NF	concanavalin A	1.0 pg mL^−1^ −100 ng mL^−1^	0.3 pg mL^−1^	[54]
	MIL-101@Au -MoS_2_ QDs	β-amyloid	10^−5^−50 ng mL^−1^	3.32 fg mL^−1^	[55]
	MoS_2_	CA19-9	0.002−50 U mL^−1^	0.25 mU mL^−1^	[56]
	MoS_2_ NSs	human epididymis-specific protein 4	10^−6^−10 ng mL^−1^	3 × 10^−7^ ng mL^−1^	[57]
Colorimetry	MoS_2_-AuNPs	CEA	0.005−10 ng mL^−1^	0.5 pg mL^−1^	[30]
	Fe-doped MoS_2_	glutathione	1–30 μM	0.577 μM	[34]
	MoS_2_@CNNS	H_2_O_2_	2.0–50.0 μM	0.02 μM	[58]
	MoS_2_/GO	glucose	1–50 μM	0.83 μM	[59]
	MoS_2_-polypyrrole-Pd	l-cysteine	1–10 μM	0.08 μM	[60]
	csDNA-Au-MoS_2_	Cd^2+^	1–500 ng mL^−1^	0.7 ng mL^−1^	[61]
	TP/SYL3C-MoS_2_	circulating tumor cells	5–10^4^ cells mL^−1^	2 cells mL^−1^	[62]
	MoS_2_/C-Au	H_2_O_2_ in living cells	1 × 10^−5^–2 × 10^−4^ M	1.82 μM	[63]
SERS	R6G-tagged MoS_2_ NF	CA19-9	5 × 10^−3^−100 IU mL^−1^	3.43 × 10^−4^ IU mL^−1^	[64]
	MoS_2_ NFs@AuNPs/MBA	CEA	0.0001−100.0 ng mL^−1^	0.033 pg mL^−1^	[65]
	Au NP@MoS_2_	cell imaging	−−	−−	[66]
Fluorescence	MoS_2_-loaded MBs	microRNA	1 pM–10 nM	10 fM	[24]
	MoS_2_ NSs	caspase-3	2−360 ng mL^−1^	0.33 ng mL^−1^	[28]
	MoS_2_	EpCAM	3–54 nM	450 pM	[67]
	MoS_2_	PSA	0–60 ng mL^−1^	0.2 ng mL^−1^	[68]
	MoS_2_	streptavidin	0–600 ng mL^−1^	0.67 ng mL^−1^	[69]
	DOX-SH/M-MoS_2_ ND	glutathione cellular imaging	0.1 × 10^−6^–100 × 10^−6^ M 0.1 × 10^−3^–4 × 10^−3^ M	30 × 10^−9^ M	[70]
	MoS_2_	ATP	0.067–26.7 μM	34.4 nM	[71]
	MoS_2_-NFP	programed cell death protein 1	125–8000 pg mL^−1^	85.5 pg mL^−1^	[72]
SPR	AuNPs-MoS_2_	miRNA-141	1–50 pM	0.5 fM	[73]

Abbreviation: toluidine blue (Tb), poly (diallyldimethylammonium chloride) (PDDA), graphene (G), polyaniline (PANI), gold@palladium nanoparticles (Au@Pt), multiwalled carbon nanotubes (MWCNTs), hepatitis B e antigen (HBeAg), trimetallic yolk-shell Au@AgPt nanocubes (Au@AgPt YNCs), carcinoembryonic antigen (CEA), dendritic platinum–copper alloy nanoparticles (DPCN), cardiac troponin I (CTnI), alpha fetoprotein (AFP), N-(aminobutyl)-N-(ethylisoluminol) (ABEI), mucin 1 (MUC1), sialic acid-binding immunoglobulin (Ig)-like lectin 5 (Siglec-5), Materials Institute Lavoisier-101 (MIL-101), concanavalin A (ConA), quantum dots (QDs), carbohydrate antigen 19-9 (CA19-9), g-C_3_N_4_ nanosheets (CNNS), polypyrrole (PPy), thymolphthalein (TP), Rhodamine 6G (R6G), 4-mercaptobenzoic acid (MBA), molecular beacons (MB), epithelial cell adhesion molecule (EpCAM), prostate specific antigen (PSA), thiolated doxorubicin (DOX-SH), adenosine triphosphate (ATP), MoS_2_ modified nanofiber paper (MoS_2_-NFP).

## Data Availability

Not applicable.
